# Utility of Red Cell Distribution Width (RDW) as a Noninvasive Biomarker for the Diagnosis of Acute Appendicitis: A Systematic Review and Meta-Analysis of 5222 Cases

**DOI:** 10.3390/diagnostics12041011

**Published:** 2022-04-17

**Authors:** Sachit Anand, Nellai Krishnan, Miro Jukić, Zvonimir Križanac, Carlos Martin Llorente Muñoz, Zenon Pogorelić

**Affiliations:** 1 Department of Pediatric Surgery, All India Institute of Medical Sciences, New Delhi 110029, India; kanusachit@gmail.com (S.A.); nellai93@gmail.com (N.K.); 2 Department of Pediatric Surgery, University Hospital of Split, 21000 Split, Croatia; mirojukic.mefst@gmail.com; 3 Department of Surgery, School of Medicine, University of Split, 21000 Split, Croatia; 4 Department of Surgery, University Hospital of Split, 21000 Split, Croatia; zvonimir.krizanac@gmail.com; 5 Surgical Clinic Medix-Muñoz, 28000 Madrid, Spain; llorentecm@gmail.com

**Keywords:** acute appendicitis, appendicitis, red cell distribution width, RDW

## Abstract

*Background*: Despite great advances in medicine, numerous available laboratory markers, and radiological imaging, the diagnosis of acute appendicitis (AA) in some cases still remains controversial and challenging for clinicians. Because of that, clinicians are still looking for an ideal marker that would be specific to AA. The red blood cell distribution width (RDW) has been recently investigated in several studies as a potential biomarker for AA. The aim of this systematic review and meta-analysis was to systematically summarize and compare all relevant data on RDW as a diagnostic biomarker for AA. *Methods*: This systematic review and meta-analysis were performed as per the Preferred Reporting Items for Systematic Reviews and Meta-Analyses (PRISMA) guidelines. Scientific databases (PubMed, Scopus, Web of Science, and Excerpta Medica database—EMBASE) were systematically searched for relevant comparative studies by two independent researches using keywords ((red cell distribution width) OR rdw) AND (appendicitis). An independent assessment of the methodological quality was performed by two authors using the Downs and Black scale. RevMan 5.4 software was used to perform the meta-analysis. *Results*: Fifteen studies were included in the final meta-analysis; the majority of the studies was retrospective. Nine studies compared the RDW values between AA and non-AA; four studies compared the same between AA and healthy controls, while two studies compared the RDW values among all three groups. The estimated heterogeneity among the studies for all outcome was statistically significant (I^2^ = 92–99%, *p* < 0.00001). The pooling the data demonstrated no statistically significant difference in the RDW values (weighted mean difference (WMD) = 0.03, 95% CI = (−0.46, 0.52), *p* = 0.91) between AA and healthy controls as well as between AA and non-AA cases (WMD = 0.23, 95%CI = (–0.19, 0.65), *p* = 0.28). A separate subanalysis was performed to evaluate the utility of this biomarker for the pediatric age group. Pooling the data demonstrated no significant difference among the AA and non-AA groups in terms of the RDW values (WMD = 0.99, 95% CI = (–0.35, 2.33), *p* = 0.15). *Conclusion*: The RDW value difference demonstrated no statistically significant difference in AA versus healthy individuals and AA versus non-AA individuals. At the moment, there is no evidence of RDW utility in diagnostic testing of AA. Further research with prospective, multicenter studies and studies targeting special patient groups with a large sample size are needed in this field.

## 1. Introduction

Acute appendicitis (AA) still remains the most common cause of acute abdomen and emergency abdominal surgery [1,2]. For years, an operative management for AA has been the treatment of choice, but nonoperative treatment for uncomplicated AA has been widely reported as an alternative to an appendectomy [3,4,5,6]. A detailed medical history and proper physical examination are the basic steps in the diagnosis of AA [3]. They are usually followed by standard laboratory parameters that include complete blood count (CBC), white blood cell count (WBC), and C-reactive protein (CRP) [7]. A differential diagnosis of AA is extensive, and many clinical conditions can mimic AA [8,9]. Radiological imaging drastically improves the diagnostic accuracy of AA. Computed tomography (CT) has an estimated sensitivity of 95% to 97% and a specificity of 93% to 99%, but, on the other hand, exposes the patients to ionizing radiation [9,10,11]. Abdominal ultrasonography has a calculated sensitivity of 75% and a specificity of 90% and uses no ionizing radiation, but it is highly operator-dependent [9,11,12]. Ultrasonography is defined as an effective first-line diagnostic tool for AA, and CT should be performed for patients with inconclusive sonographic findings [11].

In order to reduce the time needed to establish the diagnosis of AA and the number of inappropriate appendectomies, different clinical scoring systems to diagnose AA have been developed and published [9,13,14,15,16]. In addition, various laboratory parameters have been investigated to support the diagnosis of AA or/and complication of AA. These include CRP, procalcitonin, fibrinogen, matrix metalloproteinase-9, D-dimers, tumor necrosis factor alpha, interleukin 2 and 6, granulocyte colony-stimulating factor, serum amyloid A, chemokine ligand-8, bilirubin level, sodium level, etc. [17,18,19,20,21,22]. 

None of the investigated parameters have high specificity with high sensitivity for diagnosing AA [17,18,19,20,21,22]. Up until now, the timely and correct diagnosis of AA remains elusive, controversial, and challenging because symptoms of AA may be nonspecific, and the presentation can be deferrable, especially in younger children [2,23,24]. Even with all the mentioned diagnostic tools, the rate of correct diagnosis remains inadequate [25,26,27,28]. Up until now, no ideal biomarker has been identified for diagnosing AA with high specificity and sensitivity. 

The size variation measurement of circulating erythrocytes is known as the red blood cell distribution width (RDW). It is a part of the standard parameter in the automated laboratory CBC panel [29]. Several studies showed that increased values of RDW can be seen in various pathological conditions such as inflammatory bowel disease, celiac disease, pulmonary embolism, and coronary artery disease. Recent studies showed that RDW may be increased in patients with AA [30,31,32]. However, a consensus statement on the utility of RDW as a noninvasive biomarker for the diagnosis of AA is lacking.

The aim of this systematic review and meta-analysis was to systematically summarize and compare all relevant data on RDW as a diagnostic biomarker for AA.

## 2. Materials and Methods

### 2.1. Search Strategy

This systematic review and meta-analysis were performed as per the Preferred Reporting Items for Systematic Reviews and Meta-Analyses (PRISMA) guidelines [33]. Two investigators (S.A. and Z.P.) independently searched PubMed, Excerpta Medica database (EMBASE), Web of Science, and Scopus databases on 2 March 2022. The search keywords used were ((red cell distribution width) OR rdw) AND (appendicitis). The total search records were identified, and duplications were removed (Appendix A, Table A1). Subsequently, the eligibility criteria were applied to screen the studies.

### 2.2. Eligibility Criteria

The inclusion criteria were all patients (of any age) with acute AA who were diagnosed by clinico-radiological criteria and operative histopathology. RDW was compared between AA versus nonacute appendicitis (non-AA) patients and AA versus healthy controls. The non-AA patients included those with either negative appendectomy by pathology or with nonspecific abdominal pain. In scenarios where AA cases were divided into complicated and noncomplicated, RDW values of noncomplicated cases were selected for the analysis

All comparative studies with incomplete data or where the outcomes of interest were not reported were excluded. Studies on pregnant patients and those comparing only the severity/stage of appendicitis with RDW were excluded. Case reports, literature reviews, conference abstracts, commentaries, editorials, and opinion articles were also excluded. 

### 2.3. Data Extraction

Search results were obtained by two independent researchers (N.K. and M.J.). Extracted information: first author’s name, publication year, sample size, gender, age, and mean ± standard deviation (SD) of RDW in cases and controls. Disagreements were settled through discussion and consensus with a senior author (Z.P.). Data synthesis was independently performed by two investigators (Z.K. and C.M.L.M.) using the Microsoft Excel spreadsheets, version 15.24.

### 2.4. Methodological Quality Assessment

An independent assessment of the methodological quality was performed by two authors (S.A. and N.K.) using the Downs and Black scale [34]. This validated 27-point scale has four domains of assessment with minimum and maximum scores of 0 and 32, respectively. On the basis of these scores, the risk of bias was graded as high (0–15), moderate (16–23), or low (score > 23). The kappa statistics were used to identify the level of interobserver agreement regarding the risk of bias [35]. The degree of agreement was graded as slight (0.00–0.20), fair (0.21–0.40), moderate (0.41–0.60), substantial (0.61–0.80), and almost perfect (0.81–1.00).

### 2.5. Statistical Analysis

RevMan 5.4 (Cochrane Collaboration, London, UK) software was used to perform the meta-analysis. All numerical data were depicted as mean ± SD. The precision for RDW was presented with 95% confidence intervals (CIs). As the study outcomes were continuous data, mean differences (MD) were calculated for them. Subsequently, the inverse variance (IV) method was used to calculate the weighted mean difference (WMD). The I^2^ statistics were applied for the analysis of heterogeneity. In cases of significant heterogeneity (>50%), the random effects model was used. A value of *p* < 0.05 was considered as statistically significant. The guidelines from the Cochrane handbook were followed during the course of this study [36].

## 3. Results

### 3.1. Study Characteristics

**Figure 1:**
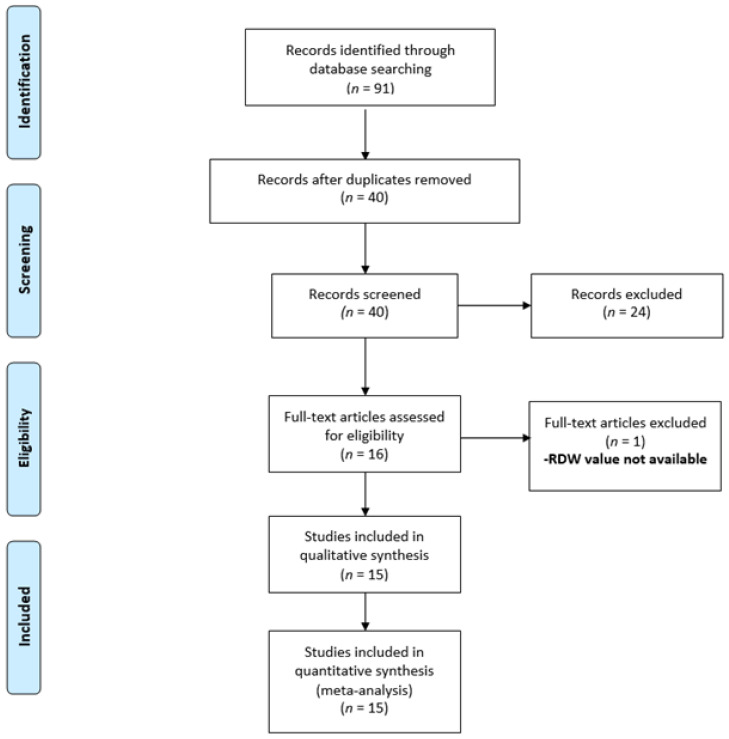
Selection of the relevant studies using the PRISMA flow diagram.

Ninety-one records were identified with our search strategy. After removal of fifty-one duplicates, forty articles were screened for eligibility. Of these, twenty-four abstracts were excluded, and sixteen full texts were assessed for inclusion (Figure 1). One of them did not mention the RDW values and was further excluded. Finally, fifteen studies were included in the final meta-analysis [3,30,31,32,37,38,39,40,41,42,43,44,45].

The study designs of these studies (Table 1) were retrospective (*n* = 13), cross-sectional (*n* = 1), and prospective study (*n* = 1). Nine studies compared the RDW values between AA and non-AA; four studies compared the same between AA and healthy controls, while two studies compared the RDW values among all three groups. A total of 5222 subjects comprising of 3575 with AA, 983 with non-AA, and 664 healthy controls were included in the meta-analysis. The baseline characteristics of the included studies are summarized in Table 1.

### 3.2. Methodological Quality Assessment

The Downs and Black scoring by two independent authors are depicted in Table 2. The average scores ranged from 21 to 26.5. The maximum score was assigned to the study by Antic et al. [40]. The risk of bias in these studies was low-to-moderate. The interobserver agreement was almost perfect (Kappa = 0.935, *p* < 0.0001) (Table 3).

### 3.3. Outcome Analysis

#### 3.3.1. RDW Values among the AA Group vs. Healthy Controls

**Figure 2:**
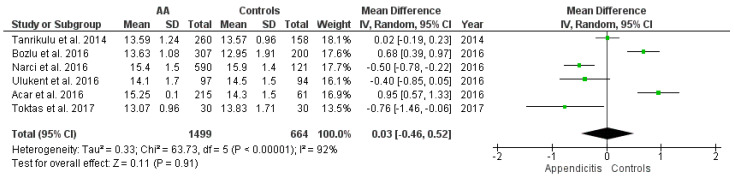
Forest plot comparison between the two patient groups (AA versus healthy controls) in terms of the average values of red cell distribution width. Abbreviations: AA—acute appendicitis; IV—inverse variance; and CI—confidence interval.

This outcome was reported by six studies [31,32,41,44,45,46]. The RDW values of a total of 1499 patients with AA and 664 healthy controls were compared. The estimated heterogeneity among the studies for this outcome was statistically significant (I^2^ = 92%, *p* < 0.00001). Pooling the data (Figure 2) using the random effects model demonstrated no statistically significant difference in the RDW values (WMD = 0.03, 95% CI = (−0.46, 0.52), and *p* = 0.91) between AA and healthy controls. 

#### 3.3.2. RDW Values among the AA vs. Non-AA Group

**Figure 3:**
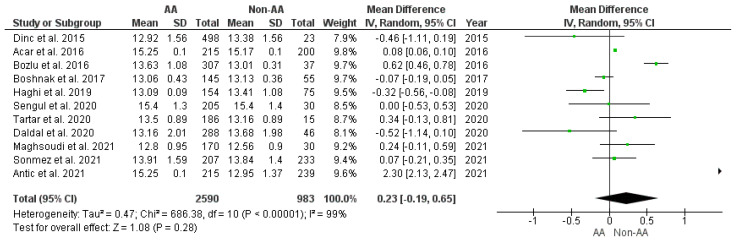
Forest plot comparison between the two patient groups (AA versus non-AA) in terms of the average values of red cell distribution width. Abbreviations: AA—acute appendicitis; non-AA—non-acute appendicitis; IV—inverse variance; and CI—confidence interval.

This outcome was reported by 11 studies [3,30,32,37,38,39,40,42,43,46,47]. The RDW values of 2590 patients with AA and 983 non-AA cases were compared. The estimated heterogeneity among the studies for this outcome was statistically significant (I^2^ = 99%, *p* < 0.00001). Pooling the data (Figure 3) using the random effects model demonstrated no statistically significant difference in the RDW values (WMD = 0.23, 95% CI = (−0.19, 0.65), *p* = 0.28) between AA and non-AA cases.

#### 3.3.3. RDW Values among the AA vs. Non-AA Group in Pediatric Patients

**Figure 4:**
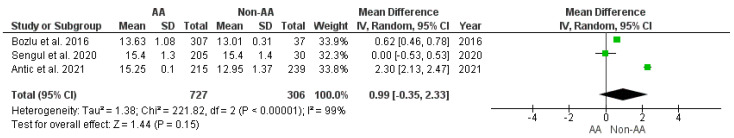
Forest plot comparison between children belonging to the AA versus non-AA groups in terms of the average values of red cell distribution width. Abbreviations: AA—acute appendicitis; non-AA—nonacute appendicitis; IV—inverse variance; and CI—confidence interval.

A separate subanalysis was performed to evaluate the utility of this biomarker for the pediatric age group. This outcome was reported by three studies only [32,39,40]. All of them compared the RDW values of AA (*n* = 727) versus non-AA (*n* = 306) cases. The estimated heterogeneity among the included studies for this outcome was statistically significant (I^2^ = 99%, *p* < 0.00001). Pooling the data (Figure 4) using a random effects model demonstrated no significant difference among the two patient groups in terms of the RDW values (WMD = 0.99, 95% CI = (−0.35, 2.33), and *p* = 0.15).

## 4. Discussion

AA is one of the most common surgical emergencies, with an incidence of approximately 100 per 100,000 people [48]. Its incidence is subject to changes according to age, geographic location, and diet [49,50]. Despite high incidence and advanced laboratory and radiologic examinations, the correct diagnosis remains challenging [51]. Although many studies speak in favor of nonsurgical treatment of uncomplicated forms of AA [4,5,6], laparoscopic appendectomy is still the standard treatment for AA in many centers worldwide [10,16,52,53,54,55]. The benefits of laparoscopic appendectomy are well known and published in numerous studies [52,53,54,55].

Finding a biomarker with the ability to diagnose AA with high specificity and high sensitivity that would easily distinguish cases of AA among the patients with nonspecific abdominal pain or those with other abdominal pathology, has been the center of attention for physicians for years. Many different parameters have been researched or are under active investigation for that purpose [51]. Establishing an easily produced, cheap, and reliable biomarker for AA could increase the correct diagnosis and reduce diagnostic delay subsequently resulting in a decreased rate of complications. The benefits of finding such biomarkers are essential, especially for a reliable differential diagnosis in rural hospitals where abdominal ultrasound and/or CT are not available and in hospitals that are the part of health systems in less developed countries. In this regard, finding the ideal biomarker would reduce the number of unnecessary appendectomies, which, despite all available diagnostic modalities, is still relatively high [52].

The RDW is a simple and automatically measured level of the variability of red blood cell size. RDW values are highly reproducible and easily attainable at no additional cost to the routine CBC [56]. The RDW is a measure of variation (anisocytosis) in the size of the circulating red cells. The RDW can be calculated either as a coefficient of variation, with a reference range of 11% to 16% depending on the laboratory, or less often as a standard deviation, with a reference range of 39 to 46 fL [57]. Recent studies suggest that RDW may also have several diagnostic and prognostic values in nonhematologic diseases. Studies on different populations recently suggested an increased mortality with increasing RDW levels. Felker et al. in their study found that RDW was a very strong and independent predictor of morbidity and mortality in heart failure populations [58]. The utilization of RDW for diagnosis and prognosis of pancreatitis and mesenteric ischemia has already been researched [59,60]. Initial RDW is an independent predictor of all-cause mortality in postresuscitation patients [61]. Furthermore, there are multiple studies researching RDW as a biomarker in many other conditions such as colon cancer, rheumatoid arthritis, Hepatitis B, and celiac disease. [56,62,63,64].

The aim of this systematic review and meta-analysis was to systematically summarize and compare all relevant data on RDW as a diagnostic biomarker for AA. To our knowledge, this is the first systematic review and meta-analysis to explore the diagnostic value of RDW in patients with AA. After selecting fifteen studies, a total of 5222 subjects were included in the meta-analysis. The estimated heterogeneity among the studies for all outcomes was statistically significant, while the risk of bias was low-to-moderate. The outcomes were measured in terms of the difference in RDW values between the AA group versus the non-AA group and the AA versus healthy control group. A separate meta-analysis was also performed to evaluate the utility of this biomarker for the pediatric age group. 

The results of this systematic review and meta-analysis demonstrated no statistically significant difference in the RDW values between AA and healthy controls and between the AA and non-AA cases. In addition, a separate subanalysis was performed as a part of our study and demonstrated no utility of this biomarker for the pediatric age group.

High heterogeneity among these studies is evident in varying findings. Narci et al. reported that adult patients with AA have RDW levels lower than healthy patients but within normal limits [31]. Haghi et al. found that the mean rate of RDW in patients with AA was lower than those with positive pathology results, and this value was lower than normal in 57.89% of those who had undergone an appendectomy [30]. Tartar et al. found a high level of RDW in complicated cases [37]. Antić et al. also showed that only WBC and RDW/MPV have diagnostic value in pediatric AA, and WBC and RDW/RBC have diagnostic value in predicting the degree of appendicitis [40]. Bozlu et al. found that RDW was higher in children with AA, while there was no difference in RDW between simple and perforated appendicitis [32]. Akbulut et al. showed that inflammatory markers, such as RDW, mean corpuscular volume (MCV), total bilirubin, CRP, neutrophil–lymphocyte ratio (NLR), platelet-to-lymphocyte ratio (PLR), and white cell nucleated (WNR) were significantly higher in patients older than 50 years old when compared to younger patients [65]. Whether these markers are useful tools for the diagnosis of AA in a high-risk vulnerable group and older patients should be investigated further. Studies have confirmed the low accuracy of these tests. Several researchers have suggested that in order to increase their sensitivity and specificity, two or more variables should be considered simultaneously in combination with WBC and CRP being the most accurate [66,67].

The results of this meta-analysis must be interpreted within the context of a few limitations. First, only seven out of fifteen studies had a low risk of bias. Second, all except two studies had a retrospective study design. Therefore, a nonuniform reporting of the study outcomes used was observed among the included studies. Third, the meta-analysis includes both children and adults. Although a separate subanalysis including only children was conducted in this study, a more homogeneous patient cohort needs to be studied in future studies. Finally, a significant heterogeneity among the included studies was observed while evaluating the above outcomes.

## 5. Conclusions

In conclusion, the RDW values demonstrate no significant different among cases with AA versus healthy controls and AA versus non-AA cases. In addition, there is no utility of RDW as a biomarker for AA in the pediatric age group. Prospective, multicenter studies with a larger sample size and studies targeting special patient groups (high-risk vulnerable groups and older patients) need to be conducted in the future before any definite conclusions in this regard are drawn.

## Figures and Tables

**Table 1:** Baseline characteristics of the included studies.

Author, Year	Study Design	Sample Size		
		AA	Non-AA	Controls
Tanrikulu et al., 2014 [45]	Retrospective	260	-	158
Dinc et al., 2015 [38]	Retrospective	498	23	-
Narci et al., 2016 [31]	Retrospective	590	-	121
Ulukent et al., 2016 [44]	Retrospective	97	-	94
Bozlu et al., 2016 [32]	Retrospective	307	37	200
Acar et al., 2016 [46]	Retrospective	215	200	61
Boshnak et al., 2017 [3]	Prospective	145	55	-
Toktas et al., 2017 [41]	Retrospective	30	-	30
Haghi et al., 2019 [30]	Retrospective	154	75	-
Tartar et al., 2020 [37]	Retrospective	186	15	-
Sengul et al., 2020 [39]	Retrospective	205	30	-
Daldal et al., 2020 [42]	Retrospective	288	46	-
Antić et al., 2021 [40]	Retrospective	223	239	-
Maghsoudi et al., 2021 [43]	Cross-sectional	170	30	-
Sönmez at al., 2021 [47]	Retrospective	207	233	-

Abbreviations: AA—acute appendicitis group. Non-AA—nonappendicitis group (consisting of cases with nonspecific abdominal pain or negative appendectomy on operative histopathology).

**Table 2:** Downs and Black scale scores for the included studies by Observer 1 and Observer 2.

Study	Reporting	External Validity	Internal Validity—Bias	Internal Validity—Confounding	Power	Total Scores
**Methodological Assessment by Observer 1**						
Tanrikulu et al., 2014 [45]	10	3	5	3	5	26
Dinc et al., 2015 [38]	10	3	5	3	0	21
Narci et al., 2016 [31]	9	3	5	3	5	25
Ulukent et al., 2016 [44]	10	3	5	3	3	24
Bozlu et al., 2016 [32]	10	3	5	3	0	21
Acar et al., 2016 [46]	10	3	5	3	2	23
Boshnak et al., 2017 [3]	10	3	5	3	1	22
Toktas et al., 2017 [41]	10	3	5	3	0	21
Haghi et al., 2019 [30]	10	3	5	3	3	24
Tartar et al., 2020 [37]	10	3	5	3	0	21
Sengul et al., 2020 [39]	9	3	5	3	0	20
Daldal et al., 2020 [42]	10	3	5	4	0	22
Antić et al., 2021 [40]	10	3	5	3	5	26
Maghsoudi et al., 2021 [43]	10	3	5	3	0	21
Sönmez at al., 2021 [47]	10	3	5	3	5	26
**Methodological Assessment by Observer 2**						
Tanrikulu et al., 2014 [45]	10	3	5	3	5	26
Dinc et al., 2015 [38]	10	3	5	4	0	22
Narci et al., 2016 [31]	10	3	5	3	5	26
Ulukent et al., 2016 [44]	10	3	5	3	3	24
Bozlu et al., 2016 [32]	10	3	5	3	0	21
Acar et al., 2016 [46]	10	3	5	4	2	24
Boshnak et al., 2017 [3]	10	3	5	4	1	23
Toktas et al., 2017 [41]	10	3	5	3	0	21
Haghi et al., 2019 [30]	10	3	5	4	3	25
Tartar et al., 2020 [37]	10	3	5	4	0	22
Sengul et al., 2020 [39]	10	3	5	5	0	22
Daldal et al., 2020 [42]	10	3	5	4	0	22
Antić et al., 2021 [40]	10	3	5	4	5	27
Maghsoudi et al., 2021 [43]	10	3	5	3	0	21
Sönmez at al., 2021 [47]	10	3	5	3	5	26

**Table 3:** The total scores and interobserver agreement (kappa statistics).

Average Scores and Interobserver Agreement					
Study	Observer 1	Observer 2	Mean	Kappa	*p*
Tanrikulu et al., 2014 [45]	26	26	26	0.935	<0.0001
Dinc et al., 2015 [38]	21	22	21.5		
Narci et al., 2016 [31]	25	26	25.5		
Ulukent et al., 2016 [44]	24	24	24		
Bozlu et al., 2016 [32]	21	21	21		
Acar et al., 2016 [46]	23	24	23.5		
Boshnak et al., 2017 [3]	22	23	22.5		
Toktas et al., 2017 [41]	21	21	21		
Haghi et al., 2019 [30]	24	25	24.5		
Tartar et al., 2020 [37]	21	22	21.5		
Sengul et al., 2020 [39]	20	22	21		
Daldal et al., 2020 [42]	22	22	22		
Antić et al., 2021 [40]	26	27	26.5		
Maghsoudi et al., 2021 [43]	21	21	21		
Sönmez at al., 2021 [47]	26	26	26		

## Data Availability

The data presented in this study are available upon request from the respective author.

## Appendix A

PubMed: ((red cell distribution width) OR rdw) AND (appendicitis)

Embase: (‘red cell distribution width’:ti,ab,kw AND appendicitis:ti,ab,kw)

Scopus: (TITLE-ABS-KEY(rdw OR ‘red AND cell AND distribution AND width’) AND TITLE-ABS-KEY (appendicitis))

Web of Science: Query 1: (ALL = (rdw)) OR ALL = (red cell distribution width)) AND Query 2: ALL = (appendicitis)

**Table A1:** Results of the search strategy.

Database	Studies
PubMed	27
EMBASE	19
Web of Science	18
Scopus	27
Total	91
Duplications	51
After duplications removed	40
